# Porcine CXCR1/2 antagonist CXCL8_(3–72)_G31P inhibits lung inflammation in LPS-challenged mice

**DOI:** 10.1038/s41598-020-57737-w

**Published:** 2020-01-27

**Authors:** Xue Wang, Yanchuan Li, Lintao Li, Zhe Jiao, Xiaoli Liu, Guofu Cheng, Changqin Gu, Xueying Hu, Wanpo Zhang

**Affiliations:** Collage of Veterinary Medicine, Huazhong Agricultural University, Wuhan, Hubei 430070 China

**Keywords:** Drug discovery, Immunology, Zoology

## Abstract

Swine pneumonia is a great threat for pig industry around the world, which is usually accompanied with neutrophils infiltration in the airway. Although interleukin-8 (CXCL8) and its receptors, CXC chemokine receptor 1 and 2 (CXCR1/2) in human have been well documented, the expression and function of CXCR1/2 is still unknown in swine. To explore the feasibility to develop new veterinary anti-inflammatory drugs targeting porcine CXCR1/2, we detected CXCR1/2 expression in swine pneumonia through Real-Time PCR and immunohistochemistry for the first time. Two porcine CXCR1/2 antagonists, CXCL8_(3–72)_N11R/G31P (pN11R) and CXCL8_(3–72)_G31P (pG31P) were prepared and their anti-inflammatory effects were evaluated using cell chemotaxis assays and animal experiments. Our data showed that CXCR1/2 expression, which was closely related to neutrophil infiltration in the lung, was significantly up-regulated in swine pneumonia. The pN11R and pG31P could effectively inhibit the directional migration of neutrophils *in vitro*. *In vivo* data also indicated that both pN11R and pG31P significantly relieved LPS-induced pneumonia in mice through decreasing the expression of *TNF-α*, *CXCL8*, and *IL-1β*, and inhibiting neutrophil influx into the lung. pG31P was more efficient. Our study suggested that it is possible to develop new veterinary anti-inflammatory drugs targeting porcine CXCR1/2, and pG31P is a promising candidate.

## Introduction

The pig industry around the word has been suffering from respiratory diseases, especially pneumonia, which caused increasing mortality and decreasing production performance^1^. Swine pneumonia usually caused by co-infection with multiple pathogens, which bring a lot of obstacles to pneumonia treatment^2,3^. Treatment of swine pneumonia nowadays mainly relies on antibiotics or vaccine. However, many countries restrict the use of antibiotics since the drug residues in food have become a serious issue^4^. Therefore, it is urgent to develop a broad-spectrum, less residual anti-inflammatory drug for pneumonia treatment.

Swine pneumonia is always accompanied with migration of neutrophils in the interstitium and broncheoalveolar space. Therefore, neutrophils are considered to play a key role in pneumonia development and rational control of neutrophils exudation and migration represent a potential therapy for swine pneumonia^5^. Previous studies indicated that neutrophils infiltration primary mediated by interleukin-8 (CXCL8) and its specific receptors, CXCR1 and CXCR2^6,7^. CXCL8 is a member of CXC chemokines, which was first discovered as a neutrophil chemotactic factor^8^. It can be can be produced by varieties of cells, such as neutrophils, monocytes, bronchial endothelial cells and airway smooth muscles cells^9–12^. CXCR1/2 belong to G-protein coupled receptor family, which are expressed on leukocytes, including neutrophil, monocytes, CD8^+^ T cells, etc.^13,14^. CXCL8 exerts its effects on neutrophils by binding with CXCR1/2. Currently, CXCL8 and CXCR1/2 have been proved to plays a central role in promoting neutrophils activation and recruitment to inflammatory site^15^. Therefore, successful blocking the interaction of CXCL8 and CXCR1/2 is a great therapy for inflammation control^16^.

During the past few decades, many antagonists target CXCL8-CXCR1/2, including Reparixin, SCH527123, SB22502 and CXCL8_(3–73)_ K11R/G31P (hG31P) were developed^17–21^. Reparixin, (R) (-)-2-(4-isobutylphenyl) propionyl methansulfonamide), is a CXCR1/2 allosteric antagonist which mainly acts on the transmembrane domain of CXCR1/2 and induces the rearrangements of 3rd and 6th α-helix, thereby preventing downstream signal transduction and inhibiting neutrophil recruitment. SCH527123, 2-hydroxy-N,N-dimethyl-3-{2-[[(R)-1-(5-methylfuran-2-yl)propyl]amino]-3,4-dioxocyclobut-1-enylamino}benzamide, is an another allosteric antagonist of CXCR1/2, which could potently bind to CXCR1/2 and are difficult to dissociate, thereby inhibiting the interaction between CXCL1 or CXCL8 with CXCR1/2, affecting downstream signal transduction and controlling neutrophil infiltration^22,23^. Several studies have shown that Reparixin and SCH527123 could relieve acute lung injury, COPD and asthma etc^19,24,25^. SB22502, (N-(2-hydroxy-4-nitrophenyl)-N′-(2-bromophenyl)urea), the first non-peptide selective inhibitor of CXCR2, was reported to inhibit CXCL8 and GRO-α-mediated Ca^2+^ mobilization, thereby reducing neutrophil chemotaxis both *in vitro* and *in vivo*. hG31P, a CXCL8 mutant which shows higher affinity to CXCR1/2 than CXCL8, could inhibit about 50% neutrophils recruitment in airway^26,27^. It was reported that hG31P has a good efficacy in the treatment of multiple inflammatory diseases, such as acute lung injury, COPD, *et al*.^18,27,28^.

It was demonstrated that CXCL8 is vital for inducing neutrophils migration to resist exogenous infection in swine^29–31^. However, the function of swine CXCR1/2 are largely unexplored. In this study, we aimed to explore the relationship between CXCR1/2 and swine pneumonia, and developed a promising anti-inflammatory drug candidate targeting porcine CXCR1/2. Our study hopes to provides a new idea for swine anti-inflammatory drugs development.

## Results

### CXCR1/2 expression in lung tissue of swine with pneumonia

**Figure 1:**
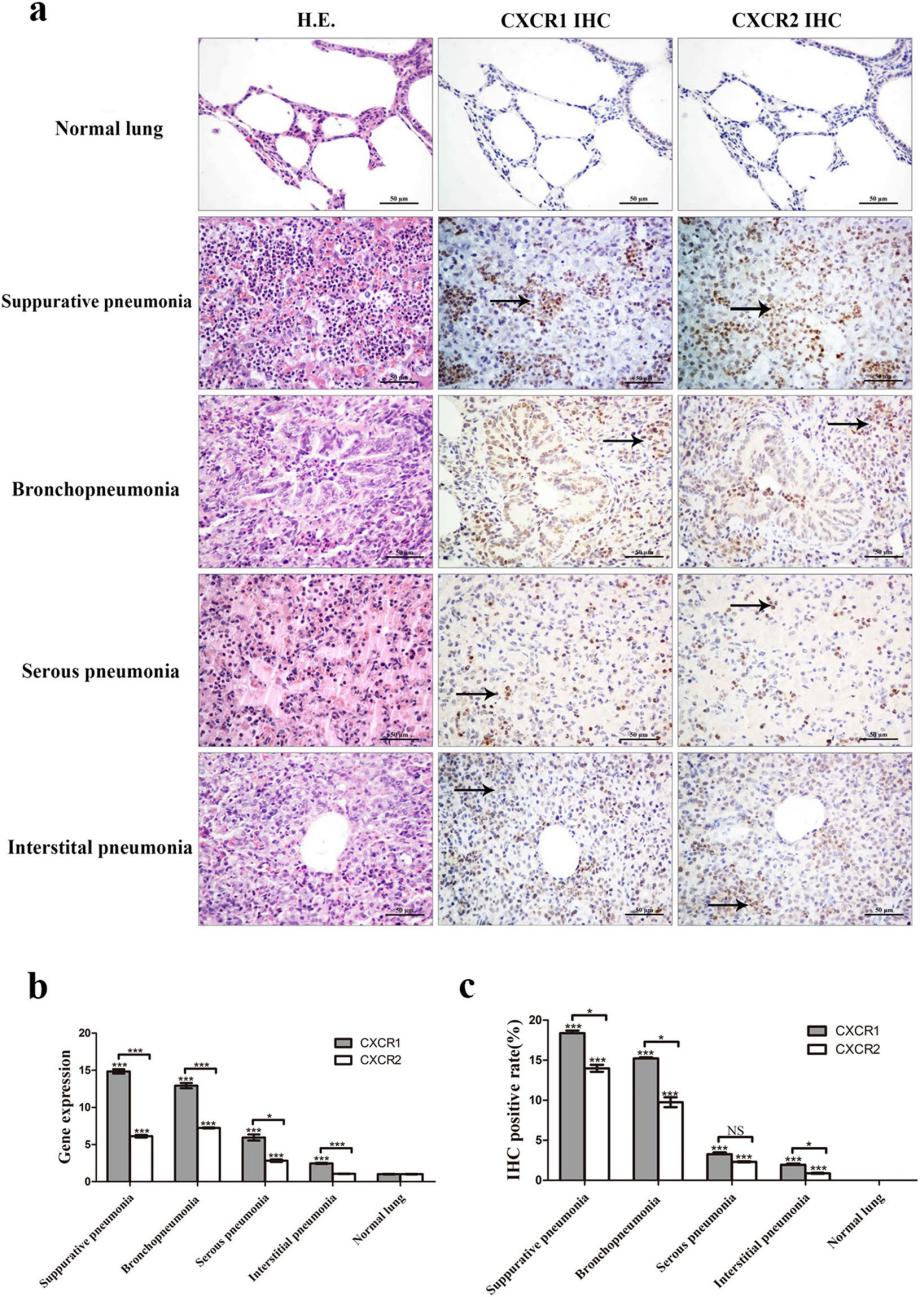
The expression of CXCR1/2 in swine pneumonia. Immunohistochemical staining showed that CXCR1/2 appeared undetectable expression in normal lung, while were positively expressed in neutrophils, which infiltrated in the lung in swine pneumonia (arrow) (**a**). Both mRNA **(b)** and protein **(c)** expression of CXCR1/2 were up-regulated in all above pneumonia, and the expression of CXCR1 was significantly higher than CXCR2 (P < 0.05). And the CXCR1/2 expression in bronchopneumonia and suppurative pneumonia are higher CXCR1/2 than that in interstitial pneumonia and serous pneumonia (P < 0.01). *P < 0.05, **P < 0.01, ***P < 0.001, unpaired t-test, two-tailed.

In this study, 127 cases of swine pneumonia were collected, including bronchopneumonia (17 cases), interstitial pneumonia (81 cases), serous pneumonia (8 cases) and suppurative pneumonia (21 cases). The alveolar septa in interstitial pneumonia were notably thickened by interstitial infiltration of numerous lymphocytes and few neutrophils. On the other hand, neutrophils infiltration in different levels in bronchioles and alveoli were observed in suppurative pneumonia, bronchopneumonia, and serous pneumonia. The expression of CXCR1 and CXCR2 was first examined by Real-Time PCR. Both CXCR1 and CXCR2 expression were up-regulated in all pneumonia samples. In addition, the expression of CXCR1 was significantly higher than CXCR2. Bronchopneumonia and suppurative pneumonia presented higher CXCR1/2 expression than interstitial pneumonia and serous pneumonia (Fig. 1b). The expression of CXCR1/2 was further examined by immunohistochemical staining of pneumonia samples. It was found that no expression of CXCR1/2 was observed in the normal lung tissues. However, the CXCR1/2 proteins were detected in neutrophils, which infiltrated in the lung in pneumonia (Fig. 1a). Additionally, the protein expression of CXCR1/2 were consistent with their mRNA expression pattern, which indicated that CXCR1/2 expression was significantly increased in swine pneumonia (Fig. 1c).

### The impact of pN11R and pG31P on neutrophil migration *in vitro*

**Figure 2:**
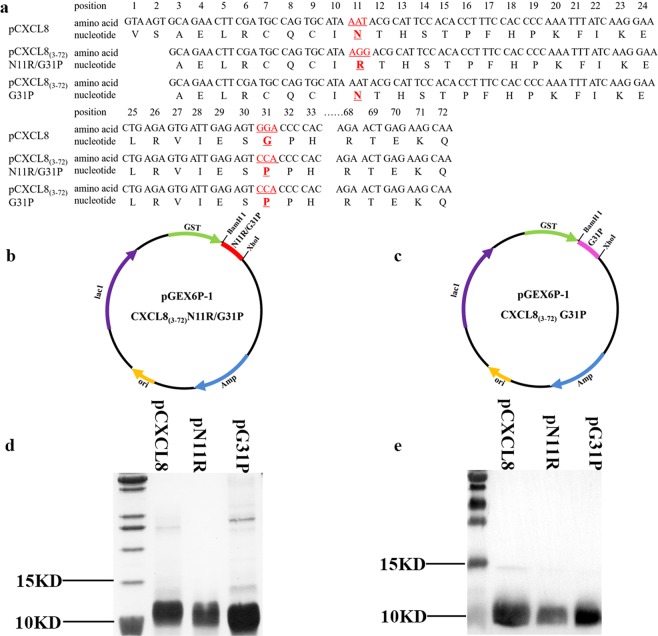
Recombinant PCXCL8_(3–72)_N11R/G31P and PCXCL8_(3–72)_G31P protein expression. The gene of porcine CXCL8_(3–72)_N11R/G31P and CXCL8_(3–72)_G31P were cloned into PGEX 6P-1, and then transformed into *E. coli* Rosetta (DE3) (**a–c**). Recombinant proteins CXCL8_(3–72)_N11R/G31P (pN11R) and CXCL8_(3–72)_G31P (pG31P) were induced by IPTG, treated with Prescission Protease, and then purified through GSTrap^TM^ FF. Two proteins molecular weight of 10KD were finally obtained (**d**). The recombinant proteins could be identified by anti-human CXCL8 antibody (**e**).

**Figure 3:**
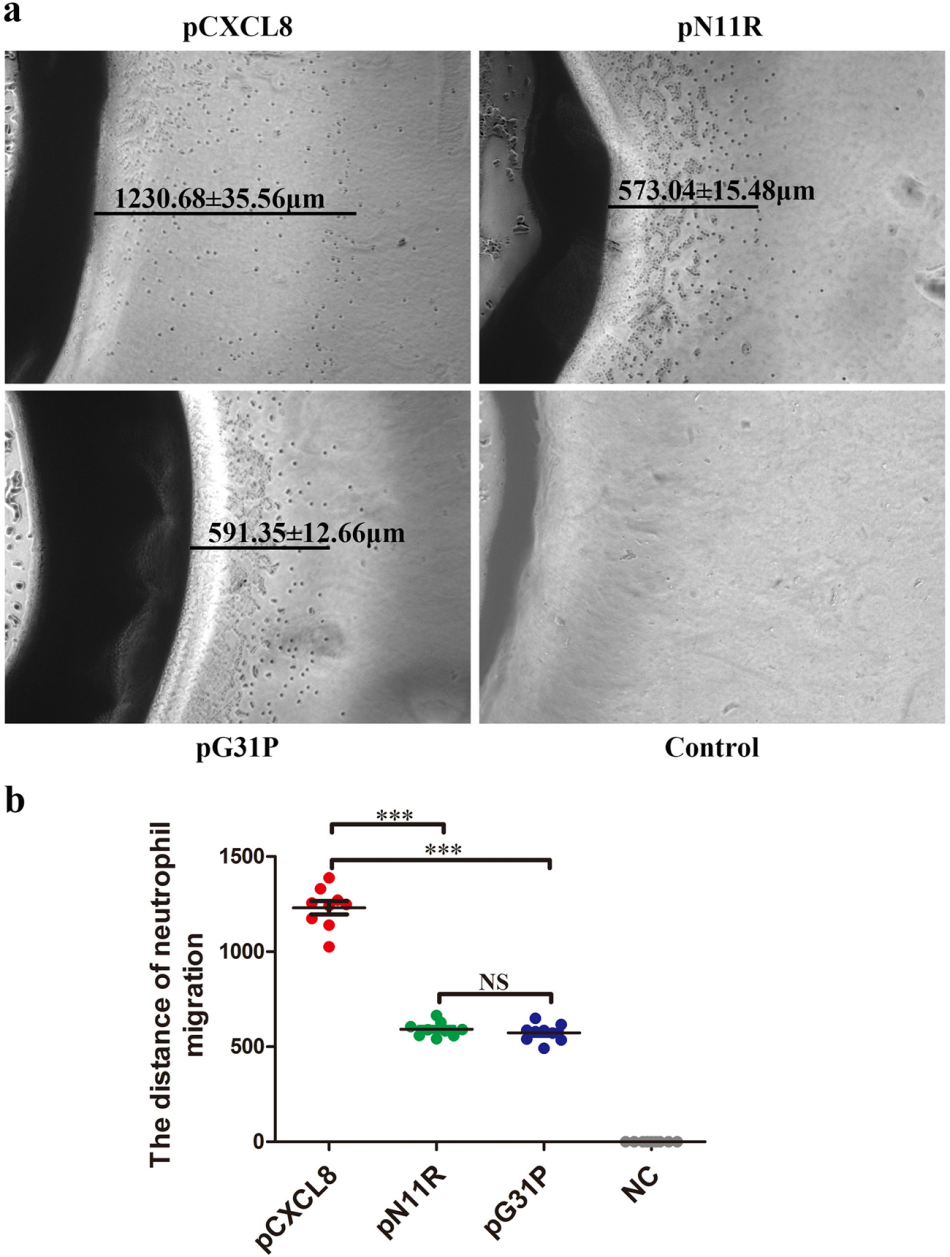
Porcine CXCR1/2 antagonist pN11R and pG31P inhibit neutrophil migration *in vitro*. The under-agarose chemotactic test was used to detect the impact of pN11R and pG31P on neutrophil migration. The mean of distance for pCXCL8 was 1230.68 ± 35.56 μm, 591.35 ± 12.66 μm for pN11R and 573.04 ± 15.48 μm for pG31P (**a**). pN11R and pG31P treatments showed a decreasing ability on neutrophil migration in *vitro* (**b**). ***P < 0.001, unpaired t-test, two-tailed.

In this study, we also expressed antagonist pN11R and pG31P targeting porcine CXCR1/2 (Fig. 2) and evaluated their chemotactic effects on neutrophil *in vitro* at first. As showed in Fig. 3a, pCXCL8, pN11R, and pG31P could induce neutrophil migration. However, the ability of pN11R and pG31P inducing neutrophil migration was decreased by about 50% compare with pCXCL8 (Fig. 3b).

### The impact of pN11R and pG31P on LPS-induced mouse pneumonia

**Figure 4:**
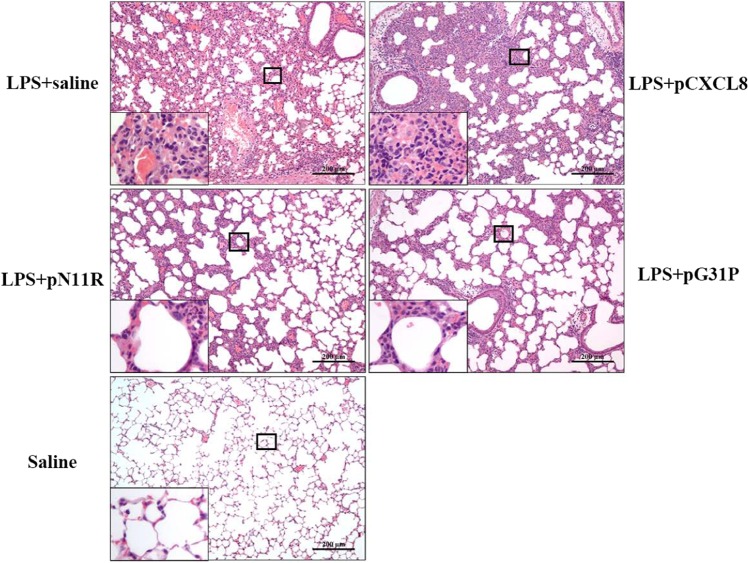
pN11R and pG31P reduce hemorrhage and neutrophils infiltration in lung of LPS-challenged mice. H.E. staining of lung from mice treated with LPS + saline, LPS + pCXCL8, LPS + pN11R, LPS + pG31P, and saline. The saline-challenged (**saline**) mice showed normal histological appearance of lung. The LPS-challenged saline-treated mice (**LPS + saline**) showed grossly hemorrhage and an increasing number of neutrophils infiltration in the lung. Hemorrhage and neutrophils infiltration in the lung were enhanced after treated with pCXCL8 (**LPS + pCXCL8**), and inversely reduced after treated with pN11R (**LPS + pN11R**) and pG31P (**LPS + pG31P**).

**Figure 5:**
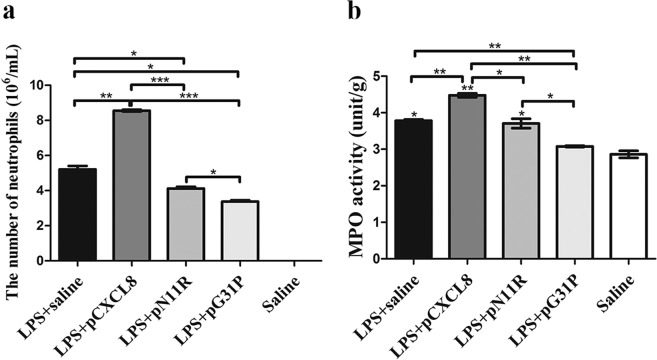
pN11R and pG31P decrease neutrophils migration and MPO activity of lung. LPS challenge significantly increased the number of neutrophils in BALF (**a**) as well as MPO (**b**) activity of lung. The neutrophils exudation and MPO activity were increased after treated with pCXCL8 (LPS + pCXCL8), and significantly reduced after treated with pN11R (LPS + pN11R) and pG31P (LPS + pG31P). *P < 0.05, **P < 0.01, ***P < 0.001, unpaired t-test, two-tailed.

**Figure 6:**
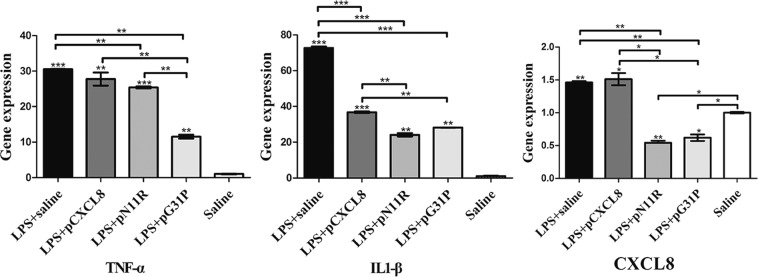
pN11R and pG31P reduce inflammatory factors expression. LPS-challenged mice (LPS + saline) appeared increasing TNF-α, IL-1β and CXCL8 expression compared to saline-challenged mice. The expressions of TNF-α, IL-1β and CXCL8 were also enhanced after treated with pCXCL8 (LPS + pCXCL8), inversely dramatically reduced after treated with pN11R (LPS + pN11R) and pG31P (LPS + pG31P). *P < 0.05, **P < 0.01, ***P < 0.001, unpaired t-test, two-tailed.

We next assessed the impact of pN11R and pG31P on inflammatory response by using a mouse pneumonia model. After 24 h of induction, the saline-challenged mice showed normal histological appearance. However, all LPS-challenged mice suffered from pneumonia. Hemorrhage and an increased number of neutrophils infiltration in the lung were observed in LPS-challenged and saline-treated mice. Treatment with pCXCL8 enhanced hemorrhage and neutrophils infiltration in the lung, whereas treatment with pN11R and pG31P significantly reduced hemorrhage and neutrophils infiltration in the lung (Fig. 4). In addition, cells in BALF were collected and counted to determine the migration of both number and type of cells into alveoli. Almost all cells migrated into BAL were neutrophils. The neutrophils were almost invisible in BALF of saline-challenged mice, while 5.2 × 10^6^ cells for LPS-challenged mice, 8.6 × 10^6^ cells for LPS + pCXCL8-challenged mice, 4.1 × 10^6^ cells for LPS + pN11R-challenged mice 3.4 × 10^6^ cells for LPS + pG31P-challenged mice. It revealed that LPS challenge significantly increased the number of neutrophils in BAL. Treatment of LPS-challenged mice with pCXCL8 significantly increased the number of neutrophils. Conversely, treatment with pN11R/pG31P significantly reduced the number of neutrophils in BAL, although the number of neutrophils was still higher than control group (Fig. 5a). Additionally, the level of neutrophil degranulation marker, MPO, in the lung of pN11R/pG31P-treated mice was also dramatically lower than that in the saline-treated group (Fig. 5b). Since the inflammatory response is strongly related to the expression of inflammatory factors, we examined the impact of our antagonist on the expression of inflammatory factors, including TNF-α, IL-1β and CXCL8. As it was shown, treatment with pN11R or pG31P reduced the expression of TNF-α, IL-1β and CXCL8. Among them, the expression of CXCL8 in the pN11R/pG31P-treated group was even lower than that in the control group (Fig. 6).

## Discussion

Pneumonia, the main disease for swine death, has caused huge losses to swine industry for a long time. Excessive neutrophil infiltration is usually found to be the primary pathological feature of pneumonia, which always leads to lung damage^32,33^. CXCL8 has been considered as an essential chemokine for neutrophil activation and recruitment. Blocking the binding of CXCL8 to its receptors, CXCR1/2, can effectively limit neutrophils recruitment and slow down inflammatory response^34,35^. In this study, the relationship between CXCR1/2 and swine pneumonia were determined using Real-Time PCR and immunohistochemical staining. The results revealed that the expression of CXCR1/2 in swine pneumonia were up-regulated. Interestingly, the expression of CXCR1/2 in bronchopneumonia and suppurative pneumonia were significantly higher than that in interstitial pneumonia and serous pneumonia (Fig. 1b,c). Immunohistochemical results showed that CXCR1/2 were expressed in neutrophil in pneumonia (Fig. 1a). Interstitial pneumonia is a type of proliferative pneumonia that is usually caused by viral infection. It is characterized by the proliferation of type II pneumocyte and less neutrophils infiltration. Bronchopneumonia, suppurative pneumonia and serous pneumonia belong to exudative pneumonia, which are mainly generated by bacterial infections or mixed infections and characterized by increasing neutrophils infiltration^32^. Histopathologically, the degree of neutrophil infiltration in suppurative pneumonia and bronchopneumonia were more obvious than that in serous pneumonia and interstitial pneumonia. Therefore, we concluded that the expression of CXCR1/2 in pneumonia was positively correlated with the number of neutrophil infiltrated in the lung. Our results demonstrated that CXCR1/2 expression was closely associated with swine pneumonia. Numerous studies showed that the expression of CXCR1 and/or CXCR2 increased in many inflammatory disease, such as ulcerative colitis and hyperoxia-induced lung injury, so that massive neutrophils could be recruited into the site of inflammation^36,37^. Reduction of CXCR1/2 expression or inhibition of CXCR1/2 binding would be an effective approach to inhibit neutrophil recruitment and relieve inflammation^38–40^. All these studies indicated that CXCR1/2 could be potential target for swine pneumonia therapeutic. Previously, a small molecular CXCL8 antagonist, hG31P was developed by Lee *et al*.^41^. They mutated Arg residue at site 11 of the main binding site of human CXCL8 to Lys11 and Pro residue at site 31 of the first β-turn of human CXCL8 to Gly to generate hG31P. It has a higher affinity to CXCR1/2 and was able to inhibit downstream signal transduction and, therefore, limit neutrophil migration^42,43^. Furthermore, it was also reported that hG31P can effectively inhibit neutrophil recruitment and reduce pathology of lung in mice models^40,44^. Porcine CXCL8 shares 73.6% homology on amino acid with hCXCL8, and has the same ELR^+^ sequence and cysteine site with hCXCL8. In this study, we substituted Arg and Pro for Lys11 and Gly31 to prepare pN11R, substituted Pro for Gly31 to prepare pG31P (Fig. 2a). Previous evidences showed that inhibiting the migration of neutrophils into the lung is critical in limiting lung inflammation^45^. Thereby, we firstly determined the impact of pN11R and pG31P on neutrophil migration *in vitro*. It was found that pN11R and pG31P could dramatically inhibit neutrophil migration *in vitro* individually. In addition, the *in vivo* effect on neutrophil migration response to LPS was also evaluated with the LPS-challenged mouse model. Histopathological results showed lower neutrophil infiltration in the lung of pN11R/pG31P-treated mice. MPO activity and neutrophils number in the BALF of LPS-challenged mice were significantly elevated compared to the control group. Treatment with pCXCL8 enhanced MPO activity and neutrophils migration in the lungs of LPS-challenged mice. By contrast, treatment with pN11R and pG31P reduced MPO activity and neutrophil migration. These results suggested that pN11R and pG31P could down-regulate neutrophil migration, therefore, contribute to limiting lung inflammation.

In addition, LPS stimulated the expression of TNF-α, IL-1β and CXCL8, however, treatment with pN11R/pG31P significantly decreased each of them. Cytokines, including IL-1β, TNF-α and CXCL8 were good indicators of lung inflammation^46^. In cases of pneumonia, inflammatory factors released in the lung could lead to increasing vascular permeability, thus resulting in rapid recruitment of neutrophils^6^. Neutrophils could express IL-1, TNF-α and CXCL8^47^. Local secretion of these cytokines could induce more neutrophil recruitment and inflammatory mediator release^15,48^. Indeed, the levels at which neutrophils secreted these cytokines are closely related to their numbers. Decreasing expression of TNF-α, IL-1β and CXCL8 suggested slight neutrophils infiltration and inflammatory response. These demonstrated that pN11R and pG31P could reduce the expression of inflammatory mediator, thereby, inhibiting inflammatory response.

Furthermore, we also compared the different impact on neutrophil migration between pN11R and pG31P, and found that although there was no difference in inducing neutrophil migration *in vitro*, the pG31P-treated mice showed lower MPO activity, neutrophils exudation in BALF and TNF-α expression (Figs. 5a and 6), it suggested that pG31P may be a candidate for the further research of swine antagonist. Fang Li *et al*. developed hG31P based on CXCL8_(3–73)_ K11R, a high agonist of neutrophil CXCR1/2^49^. However, our research demonstrated that it was possible to reduce the activity of porcine CXCL8 on inducing neutrophil migration by only mutating Pro into Gly31, and substituting Arg for Asn11 in pN11R might not increase the anti-inflammatory effect.

In summary, our study indicated that it is possible to develop veterinary anti-inflammatory drug by targeting CXCR1/2. The pG31P, which can significantly reduce neutrophils infiltration and relieve lung inflammation, is a promising drug candidate to treat swine pneumonia.

## Materials and Methods

### Ethics statement

All animals were treated in strict accordance with the Guidelines for Laboratory Animal Use and Care, which was approved by the Laboratory Animal Monitoring Committee of Huazhong Agricultural University.

### Sample collection

Totally, 127 cases of swine pneumonia were collected in Hubei province, China from March 2017 to December 2018. The samples of lung were fixed with 10% formalin for histopathological analysis. For molecular biological analysis, samples were stored at -80 °C. The types of pneumonia were confirmed by gross and histopathological observation.

### CXCR1/2 mRNA expression

Total RNA was extracted from lung samples using TRIzol^TM^ Reagent (Invitrogen, Carlsbad, CA, U.S.A.). The cDNA of CXCR1/2 were synthesized from 1 *μ*g total RNA using Prime Script^TM^ RT Kit with gDNA Eraser (Takara, Japan). Then Real-Time PCR was performed using SYBR-Green Real-Time PCR Master Mix (TaKaRa, Japan) according to the manufacturer’s instructions. Primers used in Real-Time PCR reactions were listed in Table 1. Cycling parameters were 95 °C for 10 min; 40 cycles at 94 °C for 30 sec; 56 °C for 30 sec; 72 °C for 1 min.

**Table 1:** Primers used for Real-Time PCR.

Gene	Accession NO.	Prime sequence (5′-3′)
CXCR1 (swine)	XM_021075348.1	f-TGAACATGGCTGGTGATTCAG
		r-CAGTAGGCTTAGCAGGAAGACCA
CXCR2 (swine)	XM_021075280.1	f-GATACCCGCCACCCAGGATT
		r-AGCAGGAAGACAAGGGCATAGA
β-actin (swine)	XM_003124280.5	f-TGCGGGACATCAAGGAGAAG
		r-TAGTTTCGTGGATGCCGCAG
TNF-α (mouse)	XM_021149735.1	f-TGGCCTCCCTCTCATCAGTT
		r-TTGAGATCCATGCCGTTGGC
IL-1β (mouse)	NM_008361.4	f-ATGAAAGACGGCACACCCAC
		r-GCTTGTGCTCTGCTTGTGAG
CXCL8 (mouse)	NM_011339.2	f-ATTTCCACCGGCAATGAAGC
		r-ACTGCCTGTCAAGCTGACTT
β-actin (mouse)	NM_007393.5	f-GGCTGTATTCCCCTCCATCG
		r-CCAGTTGGTAACAATGCCATGT

### Immunohistochemistry

Since porcine CXCR1/2 specific antibodies were not commercially available, CXCR1 and CXCR2 were used individually for mouse immunization to prepare polyclonal antibodies. Ten 6-week-old female BALB/c mice were respectively immunized with 100 *μ*g of recombinant porcine CXCR1 (1–90 amino acid) and CXCR2 (1–88 amino acid) in Freund’s adjuvant (Sigma, ST Louis, MO, U.S.A.) four times at biweekly intervals. One week after the final immunization, mice were bled and sera were obtained and fractionated into IgG by a protein A agarose column (GE, Piscataway, NJ, U.S.A.)^50^. The purified sera were further detected by indirect enzyme-linked immunosorbent assay (ELISA) and western blot, and finally used to immumohistochemical staining. For immunohistochemistry, the slides were deparaffinized, rehydrated, and treated with 3% H_2_O_2_. After rinsing in PBS, the slides were blocked with normal goat serum followed by incubating with anti-CXCR1 and CXCR2 antibodies (1:100) at 4 °C overnight. After rinsing with PBS, the slides were incubated with HRP-conjugated goat anti-mouse IgG (Dako, Glostrup, Denmark) at room temperature for 40 min and then were visualized with DAB Detection Kit (GK500710, Gene Tech, Shanghai, China). Six views in different fields were randomly collected from slice under microscope (Nikon, Japan). The positive rate of each slice was analyzed by Leica Image Scope software (Wetzlar, Germany).

### Preparation of porcine CXCR1/2 antagonist

The gene of porcine CXCL8_(3–72)_N11R/G31P and CXCL8_(3–72)_ G31P (Fig. 2a) were synthesized at Sangon Biotech (Shanghai) Co., Ltd. and amplified by PCR (5′-TAGGATCCGCAGAACTTCGATGCCAGTG-3′ and 5′-CGCTCGAGTTATTGCTTCTCAGTTCTCTTCAAAAA-3′). Cycling parameters were 95 °C for 10 min; 32 cycles at 94 °C for 30 sec; 56 °C for 30 sec; 72 °C for 1 min. The PCR products were digested with *BamH I* and *Xho I* (Takara, Japan) and cloned into pGEX 6P-1. The generated plasmids were transformed into *E. coli* Rosetta (DE3) cells separately. Protein expression was induced with 0.8 mmol/L isopropy1-β-D-1-thiogalactopyranoside (IPTG) (Biosharp, Hefei, China) at 18 °C for 20 h. The bacteria were collected by low-speed centrifugation and lysed with sonicator. The soluble and insoluble fractions were separated by centrifugation at 10,000 rpm for 30 min at 4 °C and analyzed by sodium dodecy1 polyacrylamide gel electrophoresis (SDS-PAGE). The recombinant pN11R and pG31P were treated with Prescission Protease (GE, Piscataway, NJ, U.S.A.) at 4 °C for 16 h and purified through GSTrap^TM^ FF (GE, Piscataway, NJ, U.S.A.) to remove glutathione S-transferase (GST)-tag.

### Western blot analysis

The pN11R and pG31P were subjected to 15% SDS-PAGE and transferred to 0.22 *μ*m polyvinylidene fluoride membrane (PVDF). The membrane was blocked with 5% skim milk (Sigma, St. Louis, MO, U.S.A.), incubated in mouse anti-human CXCL8 antibody (CSB-PA08327A0Rb, CUSABIO, Wuhan, China) and HRP-conjugated goat-anti-rabbit IgG (CUSABIO, Wuhan, China), and visualized by ECL Western Blotting Substrate (Thermo Fisher, Waltham, MA, U.S.A.).

### Neutrophil chemotaxis assay

The under-agarose chemotactic model was performed as described previously^51^. Briefly, 3 mL 1.2% agarose solution included 50% 1 × HBSS with Ca^2+^ and Mg^2+^, 50% RPMI 1640 culture medium, and 20% heat-inactivated FBS was put in the 6-well culture plate. Once the agarose solidified, 3 wells in a straight line, 3.5 mm in diameter and 2.8 mm apart, were generated on the gel. Neutrophils were isolated from peripheral blood of healthy pigs using Porcine Neutrophil Isolation Kit (Solarbio, Beijing, China) and added into the two side wells (10 *μ*L/10^7^cells/well). The porcine CXCL8 (pCXCL8), pN11R, pG31P, and cell culture medium were added into the middle well (10 *μ*L/1 *μ*g/well) respectively. The gels were incubated at 37 °C/5% CO_2_ for 6 h, and the migration distance of neutrophil was measured under microscope (Leica, Wetzlar, Germany).

### Animal procedures

18–20 g female BALB/c mice (7-week-old), which purchased from Laboratory Animal Center of Huazhong Agricultural University (Wuhan, China), were randomly divided into 5 groups, each group of 9 mice. The mice were anesthetized by diethyl ether and held on a plate titled 45°. Mice in the control group were intranasal inoculated with 20 *μ*L normal saline. Mice in the groups 1–4 were first intranasally inoculated with 10 *μ*L (10 *μ*g/*μ*L) lipopolysaccharide (LPS) to induce pneumonia. Then, groups 1–4 were intranasal inoculated with 10 *μ*L (500 *μ*g/kg) pCXCL8, 10 *μ*L (500 *μ*g/kg) pN11R, 10 *μ*L (500 *μ*g/kg) pG31P, and 10 *μ*L normal saline respectively. After 24 h of inoculation, all mice were anesthetized with sodium pentobarbital (100 *μl*, 75 mg/m*l*). 3 mice in each group were collected lung for histopathological examination, 3 mice in each group were collected lung for molecular biological detection, and 3 mice in each group were collected bronchiolar alveolar lavage (BAL) for neutrophil count.

### Lung histopathology

Mice lung tissues was fixed in 10% formalin, embedded in paraffin, sectioned into 4 *μ*m thickness, and stained with hematoxylin-eosin (H.E.). The histopathological changes were observed and recorded under a microscope (NiKon, Tokyo, Japan).

### Bronchiolar alveolar lavage collection

The lung of mice was lavaged three times with 1 mL normal saline. Lavage fluid was mixed with red blood cell lysis buffer and centrifuged for 10 min at 1,750 g. The pellet cells were resuspended in the 1 mL normal saline. 10 *μ*L cell suspension was adhered to glass slides and stained using H.E. Meanwhile, 10 *μ*L cell suspension was added to the cell-count boards to count the number of neutrophils.

### Myeloperoxidase (MPO) assay

About 100 mg frozen lung was homogenized with 1 mL saline, and centrifuged at 12,000 rpm for 10 minutes at 4 °C. The supernatant was used for MPO activity analysis according to the instruction of Mouse Myeloperoxidase ELISA Kit (Solarbio, Beijing, China).

### Cytokine bioassay

The total RNA extraction and cDNA synthesis were described above. The levels of TNF-α, CXCL8, IL-1β were detected by Real-Time PCR with the primers listed in Table 1. The cycling parameters were 95 °C for 10 min; 40 cycles at 94 °C for 30 sec; 60 °C for 1 min.

### Statistical analysis

All data was analyzed using GraphPad Prism (San Diego, CA, U.S.A). Graph bar represent means +/− S.E.M of three replicate experiments performed in triplicate (n = 3). The significance of variability among different groups was determined by unpaired t-test, P values < 0.05 was considered to be statistically significant, and P values < 0.01 was considered to be extremely significant.

## References

[1] Opriessnig T, Gimenez-Lirola LG, Halbur PG (2011). Polymicrobial respiratory disease in pigs. Anim. Health Res. Rev..

[2] Choi YK, Goyal SM, Joo HS (2003). Retrospective analysis of etiologic agents associated with respiratory diseases in pigs. Can. Vet. J..

[3] Palzer A, Ritzmann M, Wolf G, Heinritzi K (2008). Associations between pathogens in healthy pigs and pigs with pneumonia. Vet. Rec..

[4] Reeves PT (2010). Drug residues. Handb. Expe. Pharmacol..

[5] Villarino N (2013). An acute reversible experimental model of pneumonia in pigs: time-related histological and clinical signs changes. J. Vet. Pharmacol. Ther..

[6] Kolaczkowska E, Kubes P (2013). Neutrophil recruitment and function in health and inflammation. Nat. Rev. Immunol..

[7] de Oliveira S (2013). Cxcl8 (IL-8) mediates neutrophil recruitment and behavior in the zebrafish inflammatory response. J. Immunol..

[8] Yoshimura T, Matsushima K, Oppenheim JJ, Leonard EJ (1987). Neutrophil chemotactic factor produced by lipopolysaccharide (LPS)-stimulated human blood mononuclear leukocytes: partial characterization and separation from interleukin 1 (IL 1). J. Immunol..

[9] John M (1998). Expression and release of interleukin-8 by human airway smooth muscle cells: inhibition by Th-2 cytokines and corticosteroids. Am. J. Respir. Cell Mol. Biol..

[10] Ribeiro FP (2003). mRNA expression and release of interleukin-8 induced by serum amyloid A in neutrophils and monocytes. Mediat. Inflamm..

[11] Nakamura H, Yoshimura K, Jaffe HA, Crystal RG (1991). Interleukin-8 gene expression in human bronchial epithelial cells. J. Biol. Chem..

[12] Linevsky JK (1997). IL-8 release and neutrophil activation by Clostridium difficile toxin-exposed human monocytes. Am. J. Physiol..

[13] Horuk R (2001). Chemokine receptors. Cytokine Growth Factor. Rev..

[14] D’Ambrosio D, Panina-Bordignon P, Sinigaglia F (2003). Chemokine receptors in inflammation: an overview. J. Immunol. Methods.

[15] Akdis M (2016). Interleukins (from IL-1 to IL-38), interferons, transforming growth factor beta, and TNF-alpha: Receptors, functions, and roles in diseases. J. Allergy Clin. Immunol..

[16] Bizzarri C (2006). ELR+ CXC chemokines and their receptors (CXC chemokine receptor 1 and CXC chemokine receptor 2) as new therapeutic targets. Pharmacol. Ther..

[17] White JR (1998). Identification of a potent, selective non-peptide CXCR2 antagonist that inhibits interleukin-8-induced neutrophil migration. J. Biol. Chem..

[18] Matera MG, Calzetta L, Segreti A, Cazzola M (2012). Emerging drugs for chronic obstructive pulmonary disease. Expert. Opin. Emerg. Drugs.

[19] Kaur M, Singh D (2013). Neutrophil chemotaxis caused by chronic obstructive pulmonary disease alveolar macrophages: the role of CXCL8 and the receptors CXCR1/CXCR2. J. Pharmacol. Exp. Ther..

[20] Todd CM (2016). The effects of a CXCR1/CXCR2 antagonist on neutrophil migration in mild atopic asthmatic subjects. Pulm. Pharmacol. Ther..

[21] Pawlick RL (2016). Reparixin, a CXCR1/2 Inhibitor in Islet Allotransplantation. Islets.

[22] Dwyer MP (2006). Discovery of 2-hydroxy-N,N-dimethyl-3-{2-[[(R)-1-(5- methylfuran-2-yl)propyl]amino]-3,4-dioxocyclobut-1-enylamino}benzamide (SCH 527123): a potent, orally bioavailable CXCR2/CXCR1 receptor antagonist. J. Med. Chem..

[23] Gonsiorek W (2007). Pharmacological characterization of Sch527123, a potent allosteric CXCR1/CXCR2 antagonist. J. Pharmacol. Exp. Ther..

[24] Zarbock A, Allegretti M, Ley K (2008). Therapeutic inhibition of CXCR2 by Reparixin attenuates acute lung injury in mice. Br. J. Pharmacol..

[25] Nair P (2012). Safety and efficacy of a CXCR2 antagonist in patients with severe asthma and sputum neutrophils: a randomized, placebo-controlled clinical trial. Clin. Exp. Allergy.

[26] Gordon JR (2009). Amelioration of pathology by ELR-CXC chemokine antagonism in a swine model of airway endotoxin exposure. J. Agromedicine.

[27] Gordon JR (2005). The combined CXCR1/CXCR2 antagonist CXCL8(3-74)K11R/G31P blocks neutrophil infiltration, pyrexia, and pulmonary vascular pathology in endotoxemic animals. J. Leukoc. Biol..

[28] Cao Q, Li B, Wang X, Sun K, Guo Y (2018). Therapeutic inhibition of CXC chemokine receptor 2 by SB225002 attenuates LPS-induced acute lung injury in mice. Arch. Med. Sci..

[29] Petry DB (2007). Differential immunity in pigs with high and low responses to porcine reproductive and respiratory syndrome virus infection. J. Anim. Sci..

[30] Chen Y (2012). Haemophilus parasuis infection activates the NF-kappaB pathway in PK-15 cells through IkappaB degradation. Vet. Microbiol..

[31] Chang HW (2006). Immunopathological effects of porcine circovirus type 2 (PCV2) on swine alveolar macrophages by *in vitro* inoculation. Vet. Immunol. Immunopathol..

[32] Carioto, L. *Pathologic Basis of Veterinary Disease*, 5th edition. (Elsevier LTD, Oxford, 2014).

[33] Wang J (2018). Neutrophils in tissue injury and repair. Cell Tissue Res..

[34] Russo RC, Garcia CC, Teixeira MM, Amaral FA (2014). The CXCL8/IL-8 chemokine family and its receptors in inflammatory diseases. Expert. Rev. Clin. Immunol..

[35] Lerner CA, Lei W, Sundar IK, Rahman I (2016). Genetic Ablation of CXCR2 Protects against Cigarette Smoke-Induced Lung Inflammation and Injury. Front. Pharmacol..

[36] Sue RD (2004). CXCR2 is critical to hyperoxia-induced lung injury. J. Immunol..

[37] Lee YK, Hippe-Sanwald S, Lee SC, Hohenberg H, Hwang BK (2015). Distribution of the interleukin-8 receptors, CXCR1 and CXCR2, in inflamed gut tissue. J. Pathol..

[38] Schneberger D (2015). CXCR1/CXCR2 antagonist CXCL8(3-74)K11R/G31P blocks lung inflammation in swine barn dust-instilled mice. Pulm. Pharmacol. Ther..

[39] Ha H, Debnath B, Neamati N (2017). Role of the CXCL8-CXCR1/2 Axis in Cancer and Inflammatory Diseases. Theranostics.

[40] Wei J (2013). CXCR1/CXCR2 antagonism is effective in pulmonary defense against Klebsiella pneumoniae infection. Biomed. Res. IntI..

[41] Li F, Zhang X, Gordon JR (2002). CXCL8((3-73))K11R/G31P antagonizes ligand binding to the neutrophil CXCR1 and CXCR2 receptors and cellular responses to CXCL8/IL-8. Biochem. Biophys. Res. Commun..

[42] Hammond ME (1996). Receptor recognition and specificity of interleukin-8 is determined by residues that cluster near a surface-accessible hydrophobic pocket. J. Biol. Chem..

[43] Cheng HT, Yu HY, Gordon JR, Li F, Cheng JW (2017). Effects of K11R and G31P Mutations on the Structure and Biological Activities of CXCL8: Solution Structure of Human CXCL8(3-72)K11R/G31P. Molecules.

[44] Zhao X (2010). Blockade of neutrophil responses in aspiration pneumonia via ELR-CXC chemokine antagonism does not predispose to airway bacterial outgrowth. Pulm. Pharmacol. Ther..

[45] Grommes J, Soehnlein O (2011). Contribution of neutrophils to acute lung injury. Mol. Med..

[46] Gilowska I (2014). CXCL8 (interleukin 8)–the key inflammatory mediator in chronic obstructive pulmonary disease?. Postepy. Hig. Med. Dosw. (Online).

[47] Song H, Li GW, Ye J, Qian YS (2004). Modulation of mouse neutrophil cytokine secretion by Klebsiella pneumoniae. Comp. Clin. Pathol..

[48] Faccioli LH, Souza GE, Cunha FQ, Poole S, Ferreira SH (1990). Recombinant interleukin-1 and tumor necrosis factor induce neutrophil migration “*in vivo*” by indirect mechanisms. Agents Actions.

[49] Li F, Gordon JR (2001). Il-8((3-73))K11R is a high affinity agonist of the neutrophil CXCR1 and CXCR2. Biochem. Biophys. Res. Commun..

[50] Morohashi H (1995). Expression of both types of human interleukin-8 receptors on mature neutrophils, monocytes, and natural killer cells. J. Leukoc. Biol..

[51] Xu X (2017). Adenosine effectively restores endotoxin-induced inhibition of human neutrophil chemotaxis via A1 receptor-p38 pathway. Inflamm. Res..

